# Lung cancer combined with methotrexate-associated lymphoproliferative disorder: A case report

**DOI:** 10.1016/j.ijscr.2019.05.033

**Published:** 2019-05-28

**Authors:** Atsushi Sekimura, Shun Iwai, Aika Funasaki, Nozomu Motono, Katsuo Usuda, Hidetaka Uramoto

**Affiliations:** Department of Thoracic Surgery, Kanazawa Medical University, Japan

**Keywords:** Methotrexate, Lymphoproliferative disorder, Lung cancer, MTX, LPD

## Abstract

**Introduction:**

Methotrexate (MTX)-associated lymphoproliferative disorder occurs in rheumatoid arthritis patients treated with MTX; however, patients with concomitant pulmonary lesions are rare. We present a case of lung cancer combined with MTX-associated lymphoproliferative disorder for which, for which it was necessary to differentiate these from possible pulmonary metastasis.

**Presentation of a case:**

A 72-year-old man was referred to our hospital for treatment of squamous cell carcinoma in the left upper bronchus. He was receiving oral MTX and prednisolone for rheumatoid arthritis for 15 years. However, chest computed tomography performed 1 week before surgery revealed a 1-cm-sized pulmonary nodule in the right lung. Surgical pulmonary resection of the right lung tumor revealed substantial B-cell lymphoma-type lymphoproliferative disorder. Left upper lobectomy for the squamous cell carcinoma in the left upper bronchus was performed 5 weeks after the first surgery. Chest CT performed 2 weeks after the first surgery revealed a new 1-cm-sized nodule in the lower left lung lobe. However, after discontinuing oral MTX therapy, the new lesion in the left lower lobe disappeared.

**Discussion and conclusion:**

In lung cancer patients treated with MTX for rheumatoid arthritis, MTX-associated lymphoproliferative disorder should be considered as a differential diagnosis.

## Highlights

• Methotrexate-associated lymphoproliferative disorder is known to occur in patients with rheumatoid arthritis treated with methotrexate.
• FDG-PET/CT have showed FDG uptake in the nodule of methotrexate associated lymphoproliferative disorder.
• In patients with lung cancer patients who were treated with methotrexate for rheumatoid arthritis, methotrexate-associated lymphoproliferative disorder should be considered as a differential diagnosis.

## 1 Introduction

Methotrexate (MTX)-associated lymphoproliferative disorder (MTX-LPD) is a complication stemming from LPD during rheumatoid arthritis (RA) treatment and may be associated with MTX. However, mechanisms underlying its occurrence remain unclear. Patients having both pulmonary lesions and MTX-LPD are very rare [1]. Here, we present a case of primary lung cancer combined with MTX-LPD, for which this pathology had to be distinguished from typical pulmonary metastasis. This case is reported according to the SCARE criteria [2].

## 2 Presentation of case

**Fig. 1:**
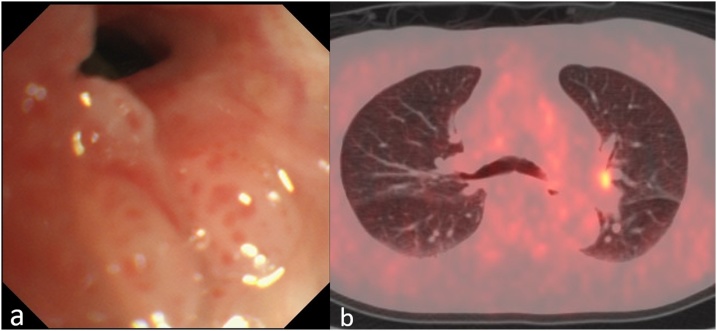
Image of squamous cell carcinoma in the left upper bronchus. [a] Bronchoscope showing tumor protrusion at the orifice of the left B1 + 2. [b] Positron emission tomography/computed tomography (PET/CT) showing high accumulation in the left B1 + 2.

A 72-year-old man was referred to our hospital for treatment of lung cancer in the left upper bronchus (Fig. 1a). He was receiving oral MTX and prednisolone for RA for 15 years. Bronchoscopy revealed a tumor protruding into the left upper bronchus; follow-up biopsy determined it to be a squamous cell carcinoma.

**Fig. 2:**
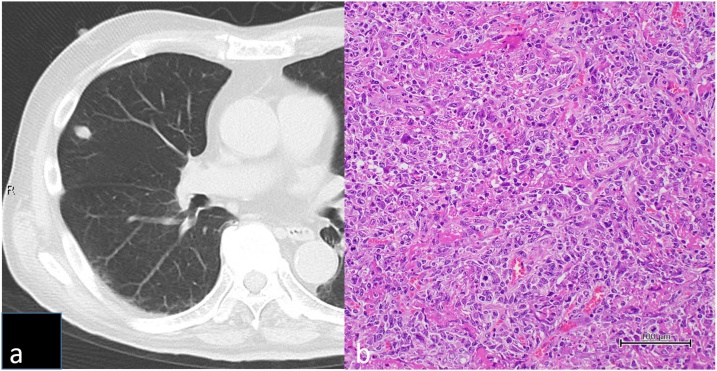
Image of the lymphoproliferative disorder in the right upper lobe. [a] Chest computed tomography 2 weeks after first surgery showing a 1-cm-sized pulmonary nodule in the contralateral lung. [b] Histopathological examination showing spindle cells with karyokinesis. Immunohistochemical staining showed that the cells were positive for CD20; a diagnosis of B-cell lymphoma-type lymphoproliferative disorder was made.

Fluorodeoxyglucose positron emission tomography (FDG-PET) and computed tomography (CT) revealed that FDG uptake in the tumor occurred only in the bronchus (Fig. 1b). However, no pulmonary nodules were found in other lung regions. Thus, we diagnosed the patient with stage IA primary lung cancer and planned a left upper lobectomy. However, chest CT performed 1 week before surgery revealed a 1-cm-sized pulmonary nodule in the contralateral lung (Fig. 2a). Although the lesion did not appear metastatic, wedge resection of the right lung nodule was performed to make a histopathologically definite diagnosis. In case the right pulmonary nodule was not a metastatic lesion, we planned radical surgery for left lung cancer. The pathological diagnosis of the right pulmonary nodule was a diffuse, large B-cell lymphoma (DLBCL), which is associated with a history of long-term oral MTX administration, and was considered a MTX-LPD-related lung lesion (Fig. 2b).

Subsequently, the oral MTX therapy was discontinued, and the patient was switched to tacrolimus for RA treatment. After 1 month, we decided to perform left upper lobectomy.

**Fig. 3:**
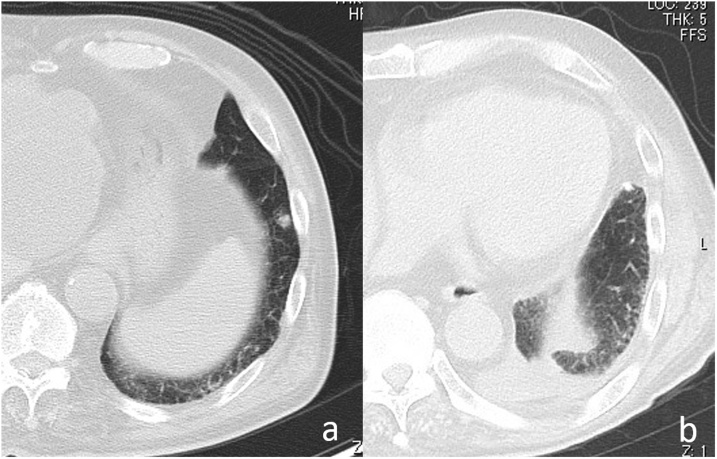
MTX-associated lymphoproliferative disorder in the left lower lobe. [a] Chest computed tomography (CT) at 2 weeks after the first surgery showing a new 1-cm-sized nodule in the left lower lobe. [b] Chest CT at 1 month after the second surgery showing that the nodules in the left lower lobe have disappeared.

Chest CT performed 2 weeks after the first surgery revealed a new 1-cm-sized nodule on the lower left lung lobe (Fig. 3a). Although FDG-PET and CT showed FDG uptake in the new nodule, the nodule could be MTX-LPD, similar to the previous nodule of the right upper lobe. We therefore planned an additional wedge resection of the left lower lobe nodule after left upper lobectomy of the lung, when the new lesion would be palpable during the operation.

Left upper lobectomy was performed 5 weeks after the first surgery. The lesion in the left lower lung lobe was not clearly palpable during the operation; however, the pleura part palpated firmly, and we performed a wedge resection. Histopathologic examination revealed no tumorigenic change. Chest CT performed 1 month after the surgery revealed that the nodules in the left lower lobe disappeared; therefore, the lesion was assumed to have disappeared at the time of the second surgery (Fig. 3b).

Postoperatively, the patient had prolonged respiratory failure owing to pulmonary function impairment. However, at 1-year postoperatively, he is alive without any relapse.

## 3 Discussion

Although MTX is an anticancer agent classified as an antifolate, it is currently widely used for treating RA. MTX-LPD occurs in patients receiving MTX. Since the first report of MTX-LPD by Elleman et al. in 1991 [3], the frequency of MTX-LPD has increased because MTX is now regularly used for treating RA, thereby making MTX-LPD a well-established disease. MTX-LPD is classified as an “other iatrogenic immunodeficiency-associated lymphoproliferative disorder” by the World Health Organization classification of tumors of hematopoietic and lymphoid tissues [4].

MTX-LPD differs from usual LPD with RA as follows: 1) the Epstein–Barr virus (EBV)-positive proportion is high in the former (27.6% vs. 9.9%); 2) there is a greater tendency for the histological DLBCL subtype in MTX-LPD than in LPD with RA (57% vs. 42.7%); 3) the onset period after RA diagnosis is slightly shorter in the former (132 vs. 240 months); and 4) LPD may initially regress after MTX discontinuation in some patients (22%) [1].

Here, we did not examine serum for the EBV antibody. However, because of long-term MTX therapy, we discontinued MTX treatment. CT performed 2 weeks after discontinuation revealed a new nodule in the contralateral lung; however, the tumor was not palpable at the second surgery performed 4 weeks after discontinuation. Furthermore, chest CT performed 1 month after the second surgery confirmed that the tumor had entirely disappeared. In our case, tumor regression could be owing to MTX discontinuation.

In this report, we diagnosed MTX-LPD pathologically by surgical resection. FDG-PET/CT showed FDG uptake in the new lesion of the left lower lobe. In the LPD lesion, as in our case, FDG uptake is usually observed [5]. Hence, distinguishing between metastatic lung tumor and MTX-LPD by imaging may be difficult. When metastatic lung cancer is found in a patient treated with MTX for RA, MTX discontinuation should be considered [6]. If the tumor does not disappear and active differential diagnosis is considered necessary, it may be wise not to hesitate to perform secondary biopsy.

The initial risk of complication of malignant tumor, including lung cancer, is higher in RA patients than in healthy individuals [7]. Furthermore, in patients with lung cancer combined with RA, the smoking rate is very high, and the odds ratio is 14.93. Smoking in RA patients increases lung cancer risk compared with that in healthy subjects [8].

Our database search could not find any report on lung cancer combined with MTX-LPD. To our knowledge, this is the first study reporting primary lung cancer with MTX-LPD. However, MTX has been widely used for treating RA for >20 years; therefore, in the future, when lymphoproliferative lung disease occurs when treating patients with lung cancer combined with RA who are using MTX, distinguishing LPD from lung cancer metastasis/recurrence may be a problem.

## 4 Conclusion

Here, we presented a case of MTX-LPD combined with lung cancer. In patients with lung cancer and RA who are using MTX, distinguishing MTX-LPD from recurrence or metastatic lesions is necessary.

## Conflicts of interest

All the authors have nothing to declare.

## Sources of funding

This research did not receive any specific grant from funding agencies in the public, commercial, or not-for-profit sectors.

## Ethical approval

I certify that this kind of manuscript does not require ethical approval by the Ethical Committee of Kanazawa Medical University.

## Consent

Written informed consent for publication of his clinical details and clinical images was obtained from the patient. A copy of the consent form is available for review by the Editor of this journal on request.

## Author contribution

Atsushi Sekimura: design, conception of the article, drafting of the article; Katsuo Usuda and Nozomu Motono: revisions, interpretation of the data; Shun Iwai and Aika Funasaki acquisition of the data and other reports; Hidetaka Uramoto: critical revisions and final approval.

## Guarantor

Hidetaka Uramoto.

## Provenance and peer review

Not commissioned, externally peer-reviewed.
